# Relapsing pneumonitis due to two distinct inhibitors of the MAPK/ERK pathway: report of a case

**DOI:** 10.1186/s12885-015-1754-3

**Published:** 2015-10-19

**Authors:** Violaine Giraud, Christine Longvert, Solène Houlle-Crepin, Claire Danel, Sylvie Labrune, Philippe Camus, Philippe Saiag, Thierry Chinet

**Affiliations:** 1 AP-HP, Department of Pulmonary Medicine and Thoracic Oncology, Hôpital A. Paré, Boulogne, & université de Versailles SQY, Boulogne, France; 2 AP-HP, Department of Dermatology, Hôpital A. Paré, Boulogne, France; 3 AP-HP, Department of Pathology, Hôpital A. Paré, Boulogne, France; 4 AP-HP, Department of Pathology, Hôpital Bichat Claude Bernard, Paris, France; 5 Department of Pulmonary Medicine, CHU Le Bocage and www.pneumotox.com, Dijon, 21079 France; 6 Service de Pneumologie et d’Oncologie Thoracique, Hôpital Ambroise Paré, 9, avenue Charles de Gaulle, Boulogne, 92 104 France

**Keywords:** Trametinib, Vemurafenib, Drug-induced pneumonitis, Granuloma, Metastatic melanoma

## Abstract

**Background:**

BRAF and MEK are component of the MAPK/ERK pathway and inhibitors of these proteins have significantly improved the outcome of metastatic melanoma. We report for the first time two sequential episodes of pneumonitis presumably induced by trametinib (a MEK inhibitor) and vemurafenib (a BRAF inhibitor) in a 50 year-old man.

**Case presentation:**

While receiving trametinib for a metastatic melanoma, the patient developed non-febrile acute respiratory failure in the context of bilateral ground-glass opacities and sub pleural reticulations on high resolution computed tomography. An excess of lymphocytes was found in the bronchoalveolar lavage fluid. Outcome was favorable after simple drug discontinuation. He subsequently developed a similar clinical-imaging picture 6 months into vemurafenib. A transthoracic lung biopsy disclosed interstitial lymphocytic infiltrate, poorly-formed granulomas with multinucleated giant cells and scattered eosinophils. Outcome was again favorable after simple drug discontinuation.

**Conclusion:**

These two episodes in the same patient suggest that MAPK/ERK inhibitors may cause interstitial lung disease and may exert cross toxicity. This side effect is of particular interest for physicians in charge of patients with melanoma but this drug family is currently under development for several other solid tumors.

## Background

The novel BRAFV600 (vemurafenib, dabrafenib) and MEK (trametinib) inhibitor are structurally-unrelated small molecules targeting two critical proteins which belong to the MAPK/ERK pathway. These drugs have transformed the prognosis of metastatic melanoma harboring BRAFV600 mutation improving both disease-free and overall survival [1–3]. We report a patient who developed trametinib and vemurafenib induced reversible pneumonitis.

## Case presentation

### Case

**Fig. 1:**
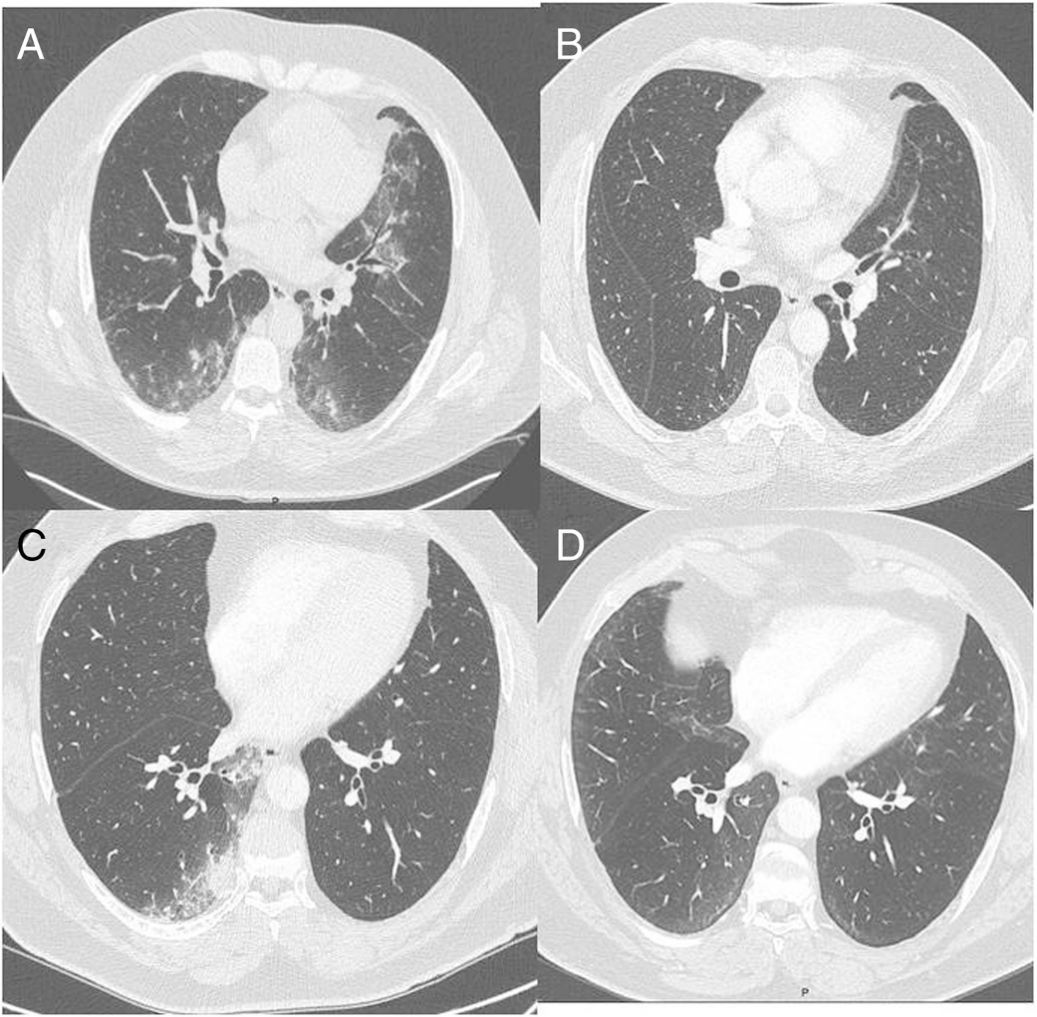
Chest CT scan (**a**): during the episode of acute respiratory failure after 5 months of treatment with trametinib, (**b**): 7 months after trametinib discontinuation and 5 months after vemurafenib initiation, (**c**): 15 months after vemurafenib initiation, (**d**): 6 months after vemurafenib discontinuation

A 50 year-old non-smoking man was diagnosed with BRAFV600E mutation-positive metastatic melanoma in 2010. Evaluation following two courses of a dacarbazine-based regimen showed progression of the cutaneous lesions and new metastases in both the lung parenchyma and mediastinal lymph nodes. After informed consent, he entered clinicaltrial.gov NCT01245062 phase III study and received trametinib 2 mg daily. Pretherapy high resolution computed tomography (HRCT) showed no evidence of interstitial lung disease (ILD). Five months into treatment, he complained of dyspnea and mild hemoptysis. There was no fever. Progressive dyspnea led to admission. Clinical examination showed bilateral crackles. Arterial blood gases results (room air) were PaO_2_ 58 mmHg, PaCO_2_ 40 mmHg, pH 7.47. On HRCT, melanoma metastases were no longer present. There were bilateral ground glass shadowing and subpleural reticulations (Fig. 1a). Blood eosinophils were 640 × 10^6^ × L^−1^. Bronchoalveolar lavage (BAL) fluid yielded 20 × 10^6^ cells.mL^−1^, of which 87 % were lymphocytes (CD4+/CD8+ ratio: 0.4) and 1 % of all cells were eosinophils. No *Pneumocystis jiroveci* (PJ) cysts were visualized. Trametinib was discontinued, while the remainder of drugs (bisoprolol, valsartan, rosuvastatin, allopurinol, paracetamol, oxazepam) was kept unchanged. Corticosteroids were not given. Patient improved clinically and radiologically (Fig. 1b).

**Fig. 2:**
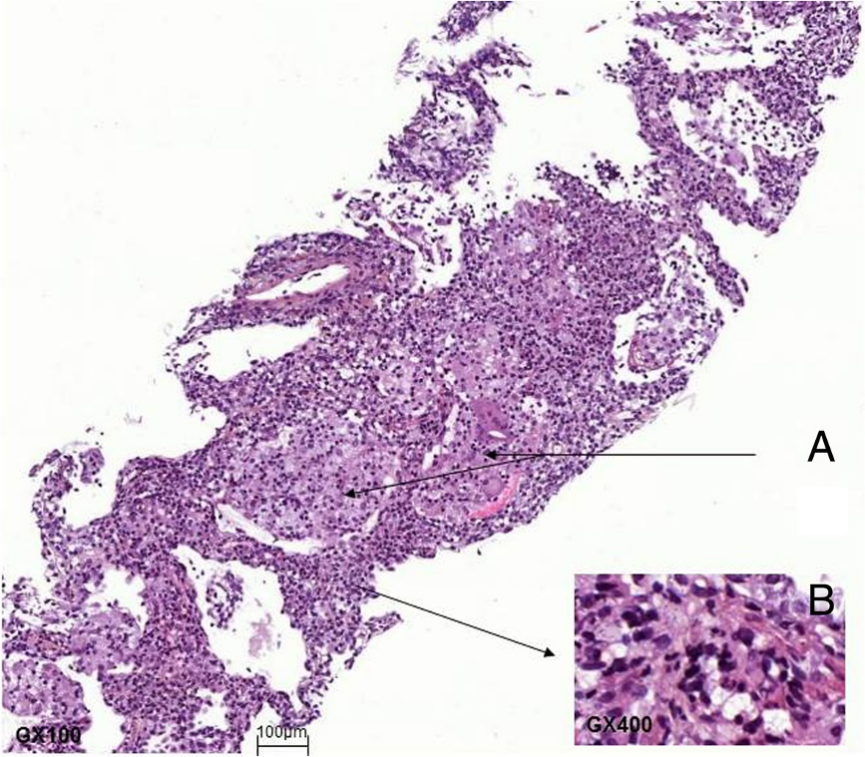
Lung biopsy. **a**: epithelioid granulomas with giant cells associated with interstitial and alveolar lymphocytic infiltrate (M: X100). **b**: inflammatory infiltrate with lymphocytes and scattered eosinophils (M: X400)

Vemurafenib (1920 mg/d) was started 2 months later. Three months into treatment, patient reported recurrence of hemoptysis and dyspnea. Although HRCT revealed new ground-glass opacities in both lungs, vemurafenib was continued. Respiratory status and HRCT slowly worsened (Fig. 1c). Fifteen months into treatment with vemurafenib, a new BAL disclosed lymphocytic alveolitis (730 × 10^3^ cells.mL^−1^, 68 % lymphocytes, CD4/CD8 ratio: 0.4, eosinophils were 3 %). There was no peripheral eosinophilia. A CT-guided transthoracic lung biopsy disclosed an interstitial and alveolar lymphocytic infiltrate, scattered eosinophils associated with poorly-formed epithelioid granulomas and multinucleated giant cells (Fig. 2). Necrosis, vasculitis, malignant cells or refractive foreign body under polarized light were absent. Special stains for PJ were negative. Polymerase chain reaction on BAL for PJ was nondiagnostic with 33.5 replication cycles indicating colonization or low burden. No anti-*Pneumocystis* drugs were given. Vemurafenib-induced pneumonitis was suspected and the drug was withheld. Respiratory symptoms progressively waned. Six months later, infiltrates and reticulations had cleared but metastatic disease progression was noted (Fig. 1d). The patient currently is on dabrafenib a BRAF inhibitor and is monitored serially and carefully as regards pulmonary signs and symptoms.

### Discussion

We report here two episodes of subacute pneumonitis that are chronologically compatible with a drug etiology. BAL findings and pathology, although not entirely specific, supported a drug reaction. Pathology findings were compatible with hypersensitivity pneumonitis to inhaled antigens, but there was no exposure to organic environmental sources. The drug etiology was also supported by improvement following drug discontinuation while corticosteroids were not given [4, 5]. Cardiogenic, infectious and neoplastic causes were considered unlikely on the basis of clinical, imaging, BAL and pathology data and because our patient’s condition improved upon drug stoppage.

Several pathological patterns have been described in patients with drug-induced pneumonitis [6]. A lymphocytic interstitial infiltrate with poorly-formed granulomas and giant cells has been described in patients with methotrexate, nitrofurantoin, BCG therapy, anti-TNF agents and mTOR inhibitor-induced lung reactions [6–8]. However, in patients with drug-induced pneumonitis, changes on pathology are rarely specific and correlate poorly with findings on imaging [9].

The present case is one of five trametinib-induced pneumonitis cases mentioned to date in the US marketing authorization file (www.accessdata.fda.gov/drugsatfdadocs/label/2014/204114s001lbl.pdf). There is no report in the literature yet. As of vemurafenib, our case is the second one described [10] but it is the first one with histological documentation. Our patient is also unique in that ILD developed while he was being treated with either one agent.

Both trametinib and vemurafenib target effectors of the MAPK/ERK pathway. Other drugs targeting upstream proteins involved in this pathway such as the tyrosine kinase inhibitors erlotinib, gefitinib, cetuximab and sorafenib may also cause pulmonary toxicity [11]. The pathophysiology of these disorders is currently unclear, but involvement of the MAPK/ERK pathway itself is one hypothesis [11].

## Conclusion

The dual inhibition of BRAF and MEK is currently one of the most promising therapeutic options to improve survival in melanoma patients [12]. Moreover, these drugs are under development for the treatment of several other solid tumors. We feel it is important to alert clinicians to the potential severe pulmonary toxicity of these two drugs targeting the MAPK/ERK pathway and to their possible class effect.

## Consent

Written informed consent was obtained from the patient for publication of this case report and any accompanying images. A copy of the written consent is available for review by the Editor of this journal.

## Abbreviations

HRCT: High Resolution Computed Tomography
ILD: Interstitial Lung Disease
BAL: Bronchoalveolar Lavage
PJ: Pneumocystis jiroveci
